# The clinical impact of late gadolinium enhancement in Takotsubo cardiomyopathy: serial analysis of cardiovascular magnetic resonance images

**DOI:** 10.1186/1532-429X-13-67

**Published:** 2011-10-29

**Authors:** Yoshihisa Naruse, Akira Sato, Kazuyuki Kasahara, Kiwa Makino, Makoto Sano, Yasuyo Takeuchi, Shiro Nagasaka, Yasushi Wakabayashi, Hideki Katoh, Hiroshi Satoh, Hideharu Hayashi, Kazutaka Aonuma

**Affiliations:** 1 Cardiovascular Division, Institute of Clinical Medicine, Graduate School of Comprehensive Human Sciences, University of 1-1-1 Tennodai, Tsukuba, Ibaraki 305-8575, Japan; 2 Department of Cardiology, Seirei Mikatahara Hospital, 3453 Mikatahara, Hamamatsu, Shizuoka 433-8575, Japan; 3 Internal Medicine III, Hamamatsu University School of Medicine: 1-20-1 Handayama, Hamamatsu, Shizuoka 431-3192, Japan

**Keywords:** Takotsubo cardiomyopathy, Cardiac magnetic resonance, Cardiac dysfunction

## Abstract

**Background:**

Our study aimed to investigate both the clinical implications of late gadolinium enhancement (LGE) by cardiovascular magnetic resonance (CMR) and the relation of LGE to clinical findings in patients with Takotsubo cardiomyopathy (TTC).

**Methods:**

We evaluated 20 consecutive patients (2 men, 18 women; median age, 77 years; interquartile range [IQR] 67-82 years) who were admitted to our hospital with the diagnosis of TTC. CMR was performed within 1 week after admission, and follow-up studies were conducted 1.5 and 6 months later.

**Results:**

In 8 patients, CMR imaging during the sub-acute phase revealed LGE in the area matched with wall motion impairment. Cardiogenic shock was more frequently observed in patients with LGE than in those without LGE (38% vs 0%, p = 0.049). The patients with LGE needed a longer duration for ECG normalization and recovery of wall motion than did those without LGE (median 205 days, IQR [152-363] vs 68 days, [43-145], p = 0.005; 15 days, [10-185] vs 7 days, [4-13], p = 0.030, respectively). In 5 of these 8 patients, LGE disappeared within 45-180 days (170, IQR [56-180]) of onset. The patients with LGE remaining in the chronic phase had higher peak creatine kinase levels than did those without LGE (median 307 IU/L, IQR [264-460] vs 202 IU/L, [120-218], p = 0.017).

**Conclusion:**

LGE by CMR in the sub-acute phase may be associated with the severity and prolonged recovery to normal of clinical findings in TTC.

## Background

Takotsubo cardiomyopathy (TTC), also referred to as transient left ventricular apical ballooning or stress cardiomyopathy, was first reported by Sato et al. [1] in 1990. Several studies have recently reported the clinical experience and profiles of this syndrome from Japan and other parts of the world [2-8]. These patients showed a reversible balloon-like left ventricular apical wall motion abnormality that returned to normal within several weeks and had chest symptoms, ST elevation, and minimal elevation of cardiac enzyme levels mimicking acute myocardial infarction without significant luminal narrowing of the coronary arteries. There was a high predominance in elderly women, and several instances were possibly triggered by physical or emotional stress. However, the pathophysiological mechanism of TTC has not yet been fully clarified.

Late gadolinium enhancement (LGE) by cardiovascular magnetic resonance (CMR) can reveal small and focal myocardial abnormalities and can be useful to diagnose various cardiac diseases [9]. It remains controversial whether LGE is present and which type of LGE is observed in patients with TTC. Initially, some studies showed that the absence of irreversible myocardial damage, as indicated by no LGE, is one of the predominant features of TTC, and CMR may be helpful in excluding myocardial infarction because LGE is not a feature of TTC [8,10-12]. However, histological analysis of the heart in TCC shows sparse foci of myocardial necrosis with contraction bands in the akinetic area [8,13-15], and recently, several studies reported LGE in patients with TTC [7,16-20]. Rolf et al. [21] showed evidence for the immunohistological basis of the LGE phenomenon in patients with TTC. Furthermore, Eitel et al. [22] found that LGE by CMR showed minute focal or patchy nonischemic myocardial scarring in 9% of patient with TTC. However, the time course of LGE by CMR and the relation between LGE and other clinical findings in TTC remain unclear. Therefore, we assessed by serial analysis both the clinical implications of LGE and the relation of LGE to clinical findings in patients with TTC.

## Methods

### Study protocol and study subjects

This study was prospectively conducted between April 2006 and November 2009 at Seirei Mikatahara Hospital, Hamamatsu, Japan. The protocol was approved by the local institutional review board, and all patients gave their written informed consent after the nature of the study and procedures had been fully explained. We prospectively enrolled 20 consecutive patients (2 men and 18 women; median age, 77 years; range 45-88 years; interquartile range (IQR) 67-82 years) who were admitted to our hospital with the diagnosis of TTC according to the criteria of the Mayo Clinic [23]. Patients were included if they had no contraindication to CMR (pacemaker, intracranial metal, claustrophobia, or obesity [> 150 kg body weight]). Coronary angiography and left ventriculography were performed within 24 hours of presentation in all patients. All patients underwent electrocardiography and transthoracic echocardiography on admission day, 3 times per week during their hospital stay, and at each outpatient clinic visit. CMR was performed within 1 week of admission, and follow-up imaging studies were conducted at 1.5 and 6 months after admission or until the findings improved. No patient was excluded for technical or image quality reasons. During follow-up, 1 patient died from a non-cardiac cause, and the remaining patients all underwent follow-up study.

### Protocol for CMR imaging

MR images were acquired with the patient in the supine position with a 1.5-T whole-body MR system (Magnetom Vision; Siemens Medical Systems, Erlangen, Germany) with a four-element phased-array cardiac coil for signal reception. The CMR images were gated to the electrocardiographic signal and were obtained during repeated breath-holds. Cine CMR images encompassing the entire left ventricular cycle were acquired on contiguous short-axis imaging planes with a segmented, fast, low-angle shot cine sequence. The CMR parameters included a repetition time of 4.2 milliseconds for each cine frame, echo time of 1.9 milliseconds, flip angle of 45°, reduction factor of 2, bandwidth of 125 kHz, section thickness of 8 mm with a 2-mm gap, field of view of 240 × 320 mm, and a 320 × 200 image matrix. Fifteen minutes after injection of a gadolinium diethylenetriamine pentaacetic acid contrast agent (Magnevist, Schering AG, Berlin, Germany; 0.1 mmol/kg), LGE inversion recovery images were acquired in the same orientation as the cine images using a 2-dimensional inversion-recovery gradient echo pulse sequence. The following imaging parameters were used: repetition time, 6.6 milliseconds; echo time, 3.1 milliseconds; flip angle, 25°; inversion time, 180 to 230 milliseconds; bandwidth, 31.25 kHz; trigger delay time for inversion recovery pulse, 200 milliseconds; section thickness, 10 mm; field of view, 240 × 320 mm; and image matrix, 192 × 256. The scan parameters were the same for all serial CMR.

### CMR image analysis

Images were analyzed according to the consensus of two observers who were blinded to patient data and the results of all other examinations. On all short-axis cine slices, the endocardial and epicardial borders were outlined manually on left ventricular end-diastolic and end-systolic images, excluding trabeculae and papillary muscles. The left ventricular ejection fraction was calculated as follows: (end-diastolic volume - end-systolic volume)/end-diastolic volume. For analysis of the LGE inversion recovery images, LGE was defined as an area of high signal intensity that was 2 and 5 standard deviations (SD) or more greater than the signal intensity of nonenhanced myocardium [22,24]. If differentiation between hyperenhanced subendocardium and the blood pool proved difficult, the subendocardial edge of the corresponding cine image was used as a reference. After determining the total volume of enhanced tissue, the percentage of LGE tissue volume for total left ventricular myocardium (LGE area ratio) was calculated as follows: (volume of enhanced tissue × 100/total volume of left ventricular myocardium) (%).

### Statistical analysis

Continuous variables are expressed as range, median, and IQR. Because of the limited sample size, non-parametric Mann-Whitney U tests were used to test for statistically significant differences in continuous variables between the study groups. Serial LGE-CMR images were compared using Wilcoxon's signed rank test for paired variables. Categorical variables were compared by Fisher's exact tests. Spearman coefficients were used to evaluate correlation between the LGE area ratio and the duration of abnormal electrocardiographic changes or echocardiographic akinesis. All probability values were 2-sided, and a value of *P *< 0.05 was considered statistically significant. All analyses were performed with the SPSS 15.0 software package (SPSS, Chicago, IL, USA).

## Results

### Clinical characteristics

Of the 20 patients with TTC, 18 (90%) were women (Table 1), of whom all were postmenopausal. Chest pain was present in 18 patients. Significant preceding emotional stress was identified in 10 patients, and acute physical stress triggered the event in 5 patients. No triggering event could be identified in 5 patients. Congestive heart failure occurred in 3 patients, and left ventricular outflow tract obstruction occurred in 1 patient. Cardiogenic shock, defined as systolic blood pressure < 80 mmHg, cool extremities, decreased urine output, and/or concomitant use of inotropes [25], occurred in 3 patients. Peak values for creatine kinase were 88-613 IU/L (205, IQR 135-225 IU/L; normal level, female < 170 IU/L, male < 225 IU/L). Serum noradrenaline levels were elevated in 11 patients (mean 601 pg/mL, IQR 418-843 pg/mL; normal level, 100-450 pg/mL).

**Table 1:** Clinical characteristics of the patients with Takotsubo cardiomyopathy

Characteristic	All	LGE [IQR]	Non-LGE [IQR]	p value
	(*n *= 20)	(*n *= 8)	(*n *= 12)	
Age (years)	77 [65-82]	75 [70-80]	78 [65-84]	0.4
Female sex, *n *(%)	18 (90%)	8 (100%)	10 (83%)	0.5
Triggering factor (emotional/physical)	15 (75%)	7 (88%)	8 (67%)	0.6
Chest pain	18 (90%)	8 (100%)	10 (83%)	0.5
Cardiac event				
CHF	3 (15%)	2 (25%)	1 (8%)	0.5
Cardiogenic shock	3 (15%)	3 (38%)	0 (0%)	0.049*
Peak creatine kinase level (IU/L)	204 [141-223]	212 [203-243]	146 [107-221]	0.09
Serum noradrenaline elevation	11 (55%)	4 (50%)	7 (58%)	1.0
ECG abnormalities	20 (100%)	8 (100%)	12 (100%)	1.0
ST elevation	12 (60%)	7 (88%)	5 (42%)	0.07
ST depression	2 (10%)	0 (0%)	2 (17%)	0.5
T inversion	10 (50%)	2 (25%)	8 (67%)	0.2
LVOT obstruction	1 (5%)	1 (13%)	0 (0%)	0.4
Ejection fraction (LVG) (%)	49 [45-53]	49 [44-52]	49 [47-53]	0.7
Typical TTC	11 (55%)	5 (63%)	6 (50%)	0.7
Time from symptom onset to CMR (days)	5 [3-6]	5 [3-6]	4 [3-6]	0.8
Ejection fraction (CMR) (%)	51 [45-54]	50 [43-53]	51 [50-58]	0.6
Duration to normalization (days)				
Electrocardiography	151 [132-188]	215 [175-363]	140 [112-151]	0.005*
Echocardiography	10 [6-14]	15 [10-18]	7 [5-13]	0.03*

Characteristics related to patient, laboratory data, ECG, and CMR in LGE (n = 8) and non-LGE (n = 12).

Values are reported as the median [interquartile range] or *n *(%).*statistically significant value (p < 0.05).

CHF indicates congestive heart failure; CMR, cardiac magnetic resonance; ECG, electrocardiography; IQR, interquartile range; LGE, late gadolinium enhancement; LVG, left ventriculogram; LVOT, left ventricular outflow tract; sBP, systolic blood pressure; and TTC, Takotsubo cardiomyopathy.

The initial electrocardiogram showed ST-segment elevation of at least 1 mm in 12 patients. Pathologic Q waves were not seen in any patient. ECG abnormalities returned to normal within 45-468 days (151, IQR 130-193 days) after onset.

Echocardiography during the acute phase revealed akinesia of the mid and/or apical portion of the left ventricle compared with hypercontraction of the basal left ventricle. Left ventricular outflow tract obstruction occurred in 1 patient. Resolution of the initial echocardiographic abnormalities was observed in all patients within 3-37 days (11, IQR 6-15 days) after onset.

**Figure 1:**
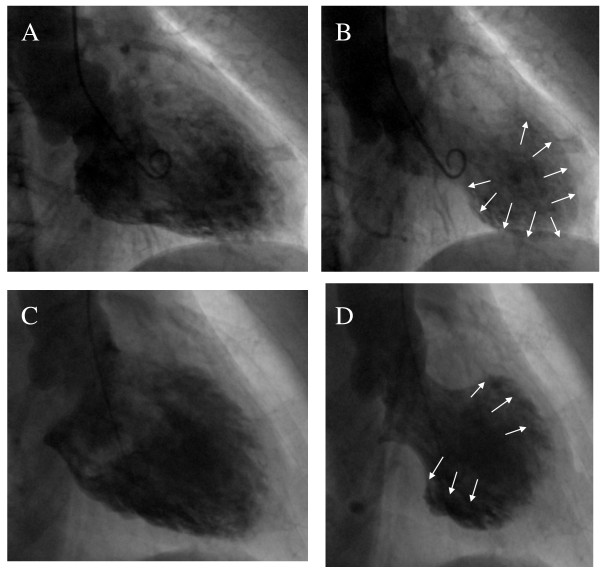
**Left ventriculography images**. *Top*, typical type of Takotsubo cardiomyopathy showing akinesis in the mid and apical portions of the LV chamber along with hypercontraction of the basal LV (A: diastole, B: systole). *Bottom*, atypical type of Takotsubo cardiomyopathy in which the LV apex is not affected (C: diastole, D: systole). LV indicates left ventricle.

Coronary angiography was normal in 16 patients, and the remaining 4 patients had only mild coronary atherosclerosis (< 50% luminal diameter stenosis). No patient had angiographic evidence of epicardial spasm. Baseline left ventricular ejection fraction was 31-61% (49, IQR 45-53%). Left ventricular akinesia on ventriculography affected the left ventricular apex and mid-portions in 11 patients, who were diagnosed as having typical TTC. The left ventricular apex was not affected in the remaining 9 patients, who were diagnosed as having atypical TTC (Figure 1).

### CMR images

CMR in the sub-acute phase was performed at 2-7 days (5, IQR 3-6 days) after onset. The left ventricular ejection fraction at this time was 41-69% (51, IQR 45-54%). Complete resolution of wall motion abnormalities had occurred in 3 patients (2 patients with typical TTC and 1 patient with the atypical form). In the remaining 16 patients, wall motion abnormalities had improved but were still demonstrable.

**Figure 2:**
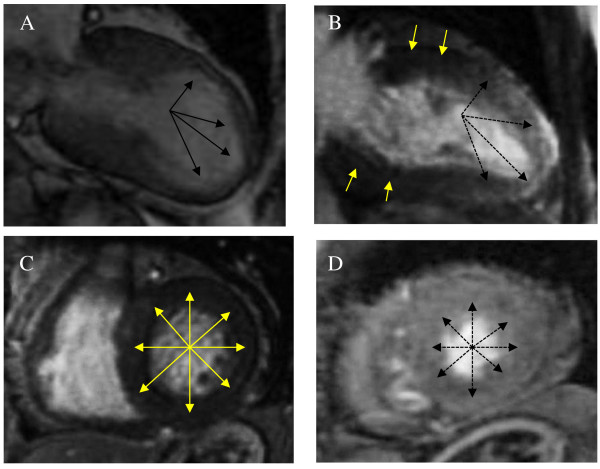
**Cardiac magnetic resonance imaging in the sub-acute phase**. (A) Cine CMR image during systole shows mid-apical dyskinesia as indicated by the arrows. (B, C, D) LGE inversion recovery images show that multiple diffuse patches were spread transmurally in the mid and apical portions, as indicated by the dotted arrows (B: vertical long axis, D: short axis at apical portion) compared with no LGE in the basal portion as indicated by the yellow arrows (C: short axis at basal portion). CMR indicates cardiac magnetic resonance and LGE, late gadolinium enhancement.

**Figure 3:**
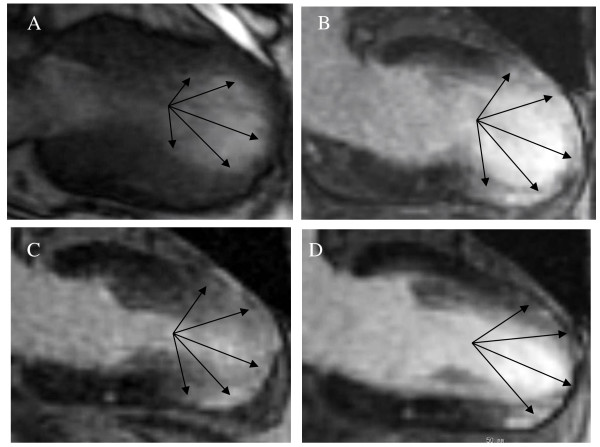
**A case of typical TTC**. (A, B) CMR images in the sub-acute phase show that the area of LGE was well matched with the area of wall motion abnormality as indicated by the arrows (A: cine CMR image during systole, B: LGE inversion recovery image). (C, D) Follow-up CMR images show that the LGE area ratio decreased gradually but remains in the chronic phase (C: 1.5 months after onset, D: 6 months after onset). CMR indicates cardiac magnetic resonance; LGE, late gadolinium enhancement; and TTC, Takotsubo cardiomyopathy.

**Figure 4:**
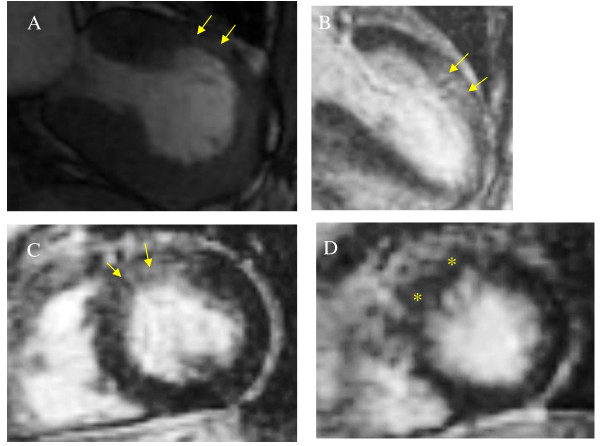
**A case of atypical TTC**. (A) Cine CMR image during systole shows mid-anterior dyskinesia as indicated by the yellow arrows. (B, C) CMR images in the sub-acute phase show that the area of LGE was well matched with the area of wall motion abnormality as indicated by the yellow arrows (B: vertical long axis, C: short axis at mid portion). (D) In follow-up CMR images of the LGE inversion recovery sequence, normal images are observed at 6 months after onset as indicated by the asterisk. CMR indicates cardiac magnetic resonance; LGE, late gadolinium enhancement; and TTC, Takotsubo cardiomyopathy.

CMR imaging revealed LGE during the sub-acute phase in 8 patients when using a threshold of 2 SD; however, none of the patients had evidence of LGE when using a threshold of 5 SD. The area of LGE was well matched with the area of wall motion abnormality (typical TTC, apical and mid portion of the left ventricle; atypical TTC, mid anteroseptal portion of the left ventricle). The signal intensity was much lower than that seen in myocardial infarction or myocarditis. LGE appeared as multiple patches that were spread transmurally (Figures 2, 3 and 4).

**Figure 5:**
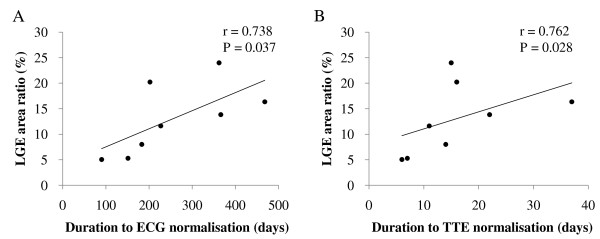
**Correlation between LGE area ratio and other clinical parameters**. Positive correlation of LGE area ratio to duration to ECG normalization (A) and duration to TTC normalization (B) was observed. LGE indicates late gadolinium enhancement and TTE, transthoracic echocardiography.

The LGE area ratio in the sub-acute phase was 5-24% (12, IQR 7-17%). A significant positive correlation was observed between the LGE area ratio in the sub-acute phase and the duration to normalization of both the electrocardiogram (r = 0.738, p = 0.037) and the echocardiogram (r = 0.762, p = 0.028, Figure 5).

In the sub-acute phase, the LGE and non-LGE subgroups did not differ significantly in age, sex, triggering factor, chest pain, congestive heart failure, peak creatine kinase, noradrenaline elevation, ECG abnormalities, left ventricular outflow tract obstruction, ejection fraction, type of TTC, and time from onset of symptoms to CMR. The LGE group had a greater prevalence of cardiogenic shock than did the non-LGE group. The LGE group in the sub-acute phase required longer time to normalize ECG and echocardiographic changes than did the non-LGE group (Table 1).

**Figure 6:**
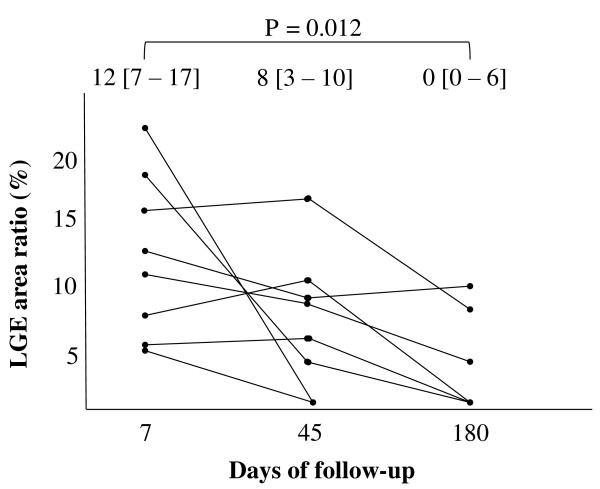
**Serial changes in the LGE area ratio in the 8 patients with LGE**. During follow-up, the LGE area ratio decreased gradually in all patients and disappeared in 5 patients at 45-180 days. Numbers at the top of the graph indicate median and interquartile range of the LGE area ratio. LGE indicates late gadolinium enhancement.

During follow-up, the LGE area ratio decreased gradually in all patients (sub-acute phase: median 12%, IQR 7-17%; 6 months after onset: median 0%, IQR 0-6%, p = 0.012). Serial CMR imaging showed complete disappearance of LGE in 5 patients at 45-180 days (170, IQR 56-180 days) after onset (Figure 6).

The 3 patients with LGE remaining in the chronic phase had higher peak creatine kinase levels than did the non-LGE patients in the chronic phase (307 IU/L, IQR 264-460 IU/L vs 202 IU/L, IQR 120-218 IU/L, p = 0.017).

## Discussion

### Major findings

The major findings of this study are as follows: (1) in 8 of 20 patients with TTC, CMR showed LGE in the matched area of wall motion impairment during the sub-acute phase; (2) cardiogenic shock was more frequently observed in the patients with LGE than in those without LGE; (3) the patients with LGE had a longer duration to ECG normalization and recovery of wall motion than did those without LGE; (4) in 5 of the 8 patients, LGE disappeared within 45-180 days (170, IQR 56-180 days) from onset of TTC; and (5) patients with LGE remaining in the chronic phase had higher peak creatine kinase levels than did patients without LGE in the chronic phase.

### CMR findings of TTC

In the present study, we showed that LGE can be present in some cases of TTC. However, the signal intensity found was lower than that usually documented in cases of myocardial infarction or myocarditis. There have been several reports of LGE in TTC so far [16-22]. Because CMR is shown to be able to detect a small and focal myocardial abnormality, LGE could reflect contraction-band necrosis. Yoshida et al. [13] reported that 3 of 9 patients had mononuclear inflammatory infiltrates, and 4 patients had contraction-band necrosis. Wittstein et al. [8] showed that 1 of 5 patients had an extensive inflammatory lymphocytic infiltrate and multiple foci of contraction-band necrosis. Both Sacha et al. [14] and Maréchaux et al. [15] reported cases of contraction-band necrosis detected at autopsy. In our study, the patients with LGE remaining in the chronic phase had higher peak creatine kinase levels than did the patients without LGE in the chronic phase. We therefore consider that contraction-band necrosis might be one cause of LGE in the patients with TTC. Our results provide evidence that the presence of LGE cannot rule out TTC.

Rolf et al. [21] found a significantly higher increase of extracellular matrix as represented by collagen-1 staining in 5 of 15 patients with LGE, which was defined as an area of high signal intensity that was 2 SD or more greater than the signal intensity of nonenhanced myocardium. Because the varying degrees of fibrosis found in several biopsies suggested the presence of islands of fibrosis in the left ventricle, this may explain the patchy character of LGE [21].

Eitel et al. [22] showed that focal and patchy LGE was detected in 22 of 239 patients when using a threshold of 3 SD instead of 5 SD above the mean of remote myocardium to define significant enhancement; however, none of their patients had evidence of LGE when using a threshold of 5 SD, which represents the cutoff for fibrosis detection in acute myocardial infarction and myocarditis. The definition of LGE in our study used the threshold of 2 SD and that may be responsible for the finding that the prevalence of LGE-positive patients was higher in our study than that in the Eitel et al. [22] study. In fact, when a threshold of 5 SD was applied to our study population, no patient had LGE in either the sub-acute phase or the chronic phase. We consider that no LGE as defined by a high threshold of 5 SD is one feature of the patients with TTC; however, we believe that evaluation of the presence of LGE using a low threshold of 2 SD might also have some clinical value, as discussed above.

Possible speculations are that severe stress-induced stunning of the apical segments leads to a patchy pattern of myocardial contraction-band necrosis possibly accompanied by a certain amount of transient focal/patchy edema or deposition of extracellular matrix resulting in LGE with low signal intensity. LGE remains in the chronic phase if the amount of contraction-band necrosis is above voxel resolution. Otherwise, LGE in the chronic phase completely normalizes if contraction-band necrosis is not present or is less than voxel resolution.

### Clinical implication

Our study showed that TTC in the sub-acute phase can have LGE with lower signal intensity than that usually documented in cases of myocardial infarction or myocarditis. For differentiation of TTC from similar symptoms of the acute coronary event, CMR can also help to define the relation of the myocardial segments with wall motion abnormality and the supplied epicardial territory. This implies that if specific CMR characteristics prove to be a non-invasive diagnostic sign for TTC, this could potentially obviate the need for an invasive diagnostic procedure.

We could also show that the patients with LGE experienced cardiogenic shock more frequently and had a longer duration to ECG normalization and recovery of wall motion than did those without LGE. These results suggest that the existence and extent of LGE in the sub-acute phase may be associated with the severity and prolonged recovery of clinical findings in TTC.

### Study limitations

This study is limited by its small sample size; thus, further studies will be needed to confirm our findings. Because we did not perform endomyocardial biopsy in this study, we cannot confirm the relation between histological findings and findings of LGE by CMR in the same patient. Although several studies with CMR have shown the presence of myocardial edema observed on T2-weighted imaging [16], T2 sequences were not acquired in our study.

## Conclusions

In conclusion, the LGE and gradual decrease in the LGE tissue volume seen in TTC are much different from the CMR findings in myocardial infarction and other cardiomyopathies and may reflect discrete myocardial contraction-band necrosis and transient focal/patchy edema or deposition of extracellular matrix. The presence of LGE cannot rule out TTC. LGE by CMR imaging in the sub-acute phase may be associated with the severity and prolonged recovery to normal of clinical findings in TTC.

## List of abbreviations used

CMR: cardiovascular magnetic resonance; IQR: interquartile range; LGE: late gadolinium enhancement; SD: standard deviations; TTC: Takotsubo cardiomyopathy.

## Competing interests

The authors declare that they have no competing interests.

## Authors' contributions

YN and AS contributed to decide the conception and design of this work and to draft, write, and revise the manuscript. KK, KM, MS, YT, SN and YW contributed to the analysis and interpretation of the data. HK, HS, HH, and KA contributed to critical revision of the manuscript for important intellectual content. All authors read and approved the final manuscript.
